# Importance of the type I insulin-like growth factor receptor in *HER2, FGFR2* and *MET*-unamplified gastric cancer with and without Ras pathway activation

**DOI:** 10.18632/oncotarget.10642

**Published:** 2016-07-17

**Authors:** Marina Saisana, S. Michael Griffin, Felicity E.B. May

**Affiliations:** ^1^ Northern Institute for Cancer Research, Newcastle upon Tyne Hospitals NHS Foundation Trust, The Medical School, University of Newcastle upon Tyne, Newcastle upon Tyne, UK; ^2^ Newcastle University Institute for Ageing, Department of Pathology, Newcastle upon Tyne Hospitals NHS Foundation Trust, The Medical School, University of Newcastle upon Tyne, Newcastle upon Tyne, UK; ^3^ Northern Oesophago-Gastric Cancer Unit, Newcastle upon Tyne Hospitals NHS Foundation Trust, The Medical School, University of Newcastle upon Tyne, Newcastle upon Tyne, UK

**Keywords:** type I IGF receptor, cell survival and proliferation, gastric cancer, KRAS-addicted, BRAF-impaired

## Abstract

Amplification of seven oncogenes: *HER2, EGFR, FGFR1, FGFR2, MET, KRAS* and *IGF1R* has been identified in gastric cancer. The first five are targeted therapeutically in patients with *HER2*-positivity*, FGFR2-* or *MET-*amplification but the majority of patients are triple-negative and require alternative strategies. Our aim was to evaluate the importance of the *IGF1R* tyrosine kinase in triple-negative gastric cancer with and without oncogenic *KRAS*, *BRAF* or *PI3K3CA* mutations. Cell lines and metastatic tumor cells isolated from patients expressed *IGF1R*, and insulin-like growth factor-1 (IGF-1) activated the PI3-kinase/Akt and Ras/Raf/MAP-kinase pathways. IGF-1 protected triple-negative cells from caspase-dependent apoptosis and anoikis. Protection was mediated *via* the PI3-kinase/Akt pathway. Remarkably, IGF-1-dependent cell survival was greater in patient samples. IGF-1 stimulated triple-negative gastric cancer cell growth was prevented by *IGF1R* knockdown and Ras/Raf/MAP-kinase pathway inhibition. The importance of the receptor in cell line and metastatic tumor cell growth in serum-containing medium was demonstrated by knockdown and pharmacological inhibition with figitumumab. The proportions of cells in S-phase and mitotic-phase, and Ras/Raf/MAP-kinase pathway activity, were reduced concomitantly. *KRAS-*addicted and *BRAF*-impaired gastric cancer cells were particularly susceptible. In conclusion, *IGF1R* and the IGF signal transduction pathway merit consideration as potential therapeutic targets in patients with triple-negative gastric cancer.

## INTRODUCTION

Gastric cancer is the third leading cause of cancer-related death worldwide. Approximately 720,000 of the 990,000 individuals diagnosed annually die from their disease [1]. Patients who present with early gastric cancer are eligible for perioperative chemotherapy and surgical resection [2]. Patients with advanced, inoperable disease are offered palliative chemotherapy to prolong survival and alleviate symptoms [3]. The median survival is only 11.2 months [4]. There is an urgent need for preclinical validation of therapeutic targets followed by clinical trials of appropriate drugs.

Approximately 7-17% of advanced gastric cancers have *HER2* overexpression or amplification [5], 2-7% have *FGFR2* amplification [6, 7] and 2-8% have *MET* amplification [8, 9]. *KRAS* is mutated in a significant proportion but *BRAF* is mutated less frequently in gastric cancers [10]. Trastuzumab is offered to, and lapatinib is in clinical trial for, patients with human epidermal growth factor receptor 2 (HER2)-positive advanced gastric cancer [11], [12], and inhibitors that target hepatocyte growth factor receptor (c-Met) and fibroblast growth factor receptors (FGFRs) are under clinical trial [13]. There remain around 70% of patients who are not eligible for targeted therapies against these three tyrosine kinase receptors. Such triple-negative cancers require alternative therapeutic strategies.

A recent genomic study of gastric cancers identified somatic copy number alterations of seven oncogenes involved in tyrosine kinase/MAP-kinase pathways: *KRAS, EGFR, HER2, FGFR1, FGFR2, MET* and *IGF1R* [14]. Activating mutations were found in *KRAS* and *PIK3CA*. A second study reported *IGF1R* amplification especially in CIN and GS subtypes [15]. Insulin-like growth factor (IGF) pathway activity has been reported in *MET*-amplified gastric cancer cells [16, 17] and the pathway has been proposed as a means by which *HER2*-amplified NCI-N87 circumvent lapatinib inhibition [18]. The IGF-dependence of triple-negative gastric cancer cells has not been investigated.

The IGF signal transduction pathway has three ligands, IGF-1, IGF-2 and insulin, which transduce their signals through the type I IGF receptor, that is encoded by *IGF1R*, and the insulin receptor [19]. Inhibitors of the IGF signal transduction pathway include monoclonal antibodies specific for the type I IGF receptor, and tyrosine kinase inhibitors most of which target both the type I IGF and the insulin receptors. Phase I and phase II clinical trials of these inhibitors were encouraging [20, 21]. Phase III trials in non-small cell lung cancer (NSCLC) failed to show significant clinical benefit [22] possibly because patients were not stratified prior to treatment [23], [24].

In the present study, the significance of *IGF1R* and the importance of the IGF signal transduction pathway in the phenotypic responses of triple-negative gastric cancer cell lines with and without mutations in *KRAS, BRAF* and *PIK3CA*, and in tumor cells isolated directly from gastric cancer patients, are investigated.

## RESULTS

### Tyrosine kinase receptor expression

**Figure 1:**
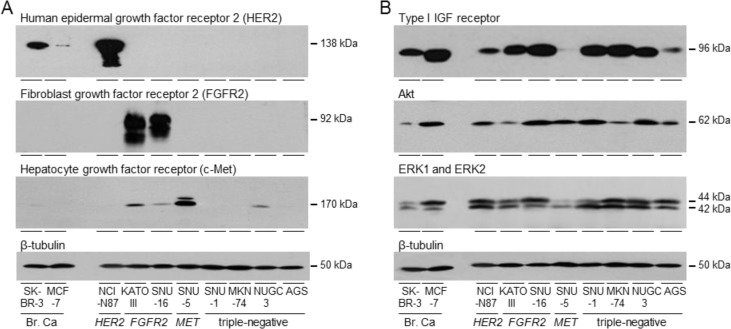
Expression of tyrosine kinase receptors in gastric cancer cells SK-BR-3, MCF-7, NCI-N87, KATO III, SNU-16, SNU-5, SNU-1, MKN74, NUGC3 and AGS were grown to 70% confluence and lysed. Aliquots of 10 μg of protein were electrophoresed on polyacrylamide gels, transferred to nitrocellulose and incubated with antibodies against HER2, FGFR2, c-Met **A.**, type I IGF receptor, pan Akt, ERK1 and ERK2 **B.** and β-tubulin and developed as described in the Materials and Methods. Representative images are shown.

HER2, FGFR2 and c-Met expression was analyzed in gastric adenocarcinoma cell lines. HER2 was expressed at higher levels in NCI-N87 than in *HER2*-amplified SK-BR-3 breast cancer cells (Figure 1A). KATO III and SNU-16 expressed highest concentrations of FGFR2. SNU-5 expressed the most c-Met, followed by KATO III and low levels were detected in SNU-16 and NUGC3. Expression of the three tyrosine kinase receptors was undetectable in SNU-1, MKN74 and AGS, and NUGC3 expressed only low levels of c-Met. These cells provide models of unamplified, triple-negative gastric cancer with which to investigate the importance of signal transduction pathways that might drive cell survival and proliferation.

Type I IGF receptor expression was measured in the gastric cancer cell lines and compared to expression in IGF-responsive breast cancer cells [25–27] (Figure 1B). Kato III, SNU-16, SNU-1, MKN74 and NUGC3 expressed high amounts of type I IGF receptor, comparable to MCF-7 breast cancer cells that are exquisitely responsive to IGFs. NCI-N87 and AGS expressed relatively less receptor and there were very low levels in SNU-5. All gastric cancer cell lines expressed Akt, ERK1 and ERK2, but Akt expression was lower in KATO III and MKN74, and ERK1 and ERK2 were lower in SNU-5.

### Activation of IGF signal transduction

**Figure 2:**
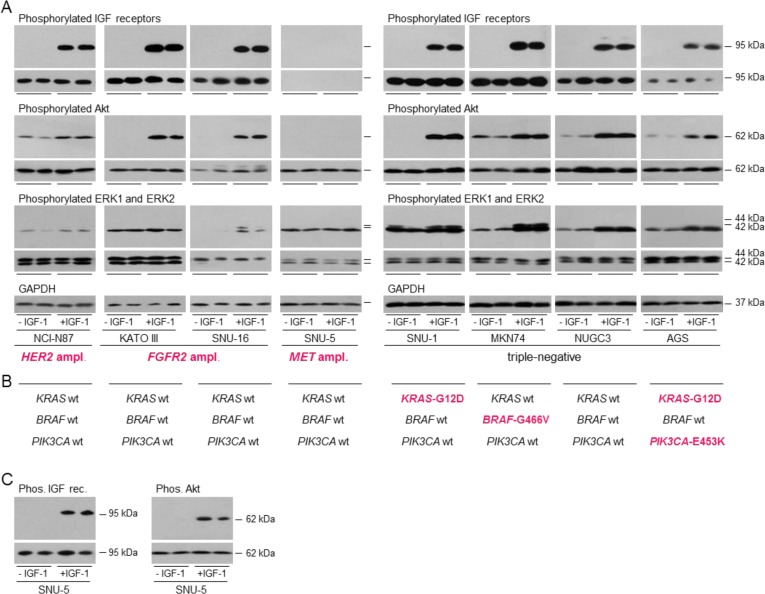
Activation of signal transduction pathways in gastric cancer cells NCI-N87, KATO III, SNU-16, SNU-5, SNU-1, MKN74, NUGC3 and AGS were withdrawn for one (SNU-1) or two days, stimulated with 50 ngml^−1^ IGF-1 for 15 min and lysed **A.** and **C.** Aliquots of 10 μg of protein were electrophoresed on polyacrylamide gels, transferred to nitrocellulose and incubated with antibodies against, phosphorylated IGF receptors, type I IGF receptor, phosphorylated Akt, pan Akt, phosphorylated ERK1 and ERK2, ERK1 and ERK2, or GAPDH and developed. Representative images from incubations with the same antibody concentrations and exposure times for each detection antibody are shown to allow comparison between cell lines. Images obtained after incubation with antibodies against the total individual proteins are beneath those of the corresponding phosphorylated proteins. Driver amplifications and mutations discussed in the text are shown in bold cerise **B.** For SNU-5, additional longer exposures after incubation with higher concentrations of the type I IGF receptor and phosphorylated IGF receptor antibodies and after incubation with the same concentration of phosphorylated Akt antibody and are shown **C.**

The implication that gastric cancer cells might be responsive to the IGF signal transduction pathway was explored. To allow the effects of growth factors to be tested, cells were cultured for one (SNU-1) or two days in withdrawal medium that contained 10% calf serum that had been incubated with dextran-coated charcoal to remove growth factors prior to stimulation with IGF-1 [25, 28]. IGF receptor phosphorylation was undetectable in cells grown in the absence of IGF-1 but was detectable after IGF-1 stimulation in all cell lines except *MET*-amplified SNU-5, which has low receptor levels (Figure 2A). IGF-stimulated receptor phosphorylation and total type I IGF receptor were detected in SNU-5 after incubation with higher antibody concentrations (Figure 2C). Phosphorylated Akt was not detected in the absence of IGF-1 in four of the cell lines, including those with amplified *FGFR2*, and was low in the other cell lines. IGF-1 stimulated Akt phosphorylation in all eight gastric cancer cell lines. In *MET*-amplified SNU-5, stimulation was not detected under the standardized detection conditions but was after prolonged exposure (Figure 2C). Stimulation of Akt phosphorylation was greatest in Kato III, SNU-1, MKN74 and NUGC3. IGF-1 stimulated ERK1 and ERK2 phosphorylation in NCI-N87, SNU-16, SNU-1, MKN-74, NUGC3 and AGS cells.

Thus the IGF signal transduction pathway is active and IGF-1 activates both the PI3-kinase/Akt and Ras/Raf/MAP-kinase pathways in gastric cancer cells. SNU-1 and AGS harbor activating *KRAS* mutations (Figure 2B) [29]; SNU-1 are addicted to the Ras pathway as evidenced by their sensitivity to the MEK1 and MEK2 inhibitor, selumetinib [29]. AGS are less sensitive to selumetinib possibly because they have an activating *PI3KCA* mutation [29]. MKN74 have a *BRAF*-impaired mutation and are insensitive to the *BRAF*-V600E-specific inhibitor, vemurafenib [30]. NUGC3 are wild-type for *KRAS, BRAF* and *PI3KCA*. Notably, IGF-1 stimulated phosphorylation of ERK1 and ERK2 in *KRAS*-activated and *BRAF*-impaired gastric cancer cells. Similarly, Akt phosphorylation was stimulated in *PI3KCA*-activated AGS cells.

### IGF-1 protects triple-negative gastric cancer cells from cell death

Anoikis is a form of programmed cell death initiated by disruption of interactions between normal epithelial cells and the extracellular matrix. Anoikis resistance enables cancer cell invasion and dissemination [31]. Anoikis is mediated through the intrinsic pathway in which caspase 3 is cleaved into active 17 and 12 kDa fragments. Consequently, 113 kDa poly(ADP-ribose) polymerase (PARP) is inactivated by removal of its DNA-binding domain to produce the 89 kDa inactive fragment.

**Figure 3:**
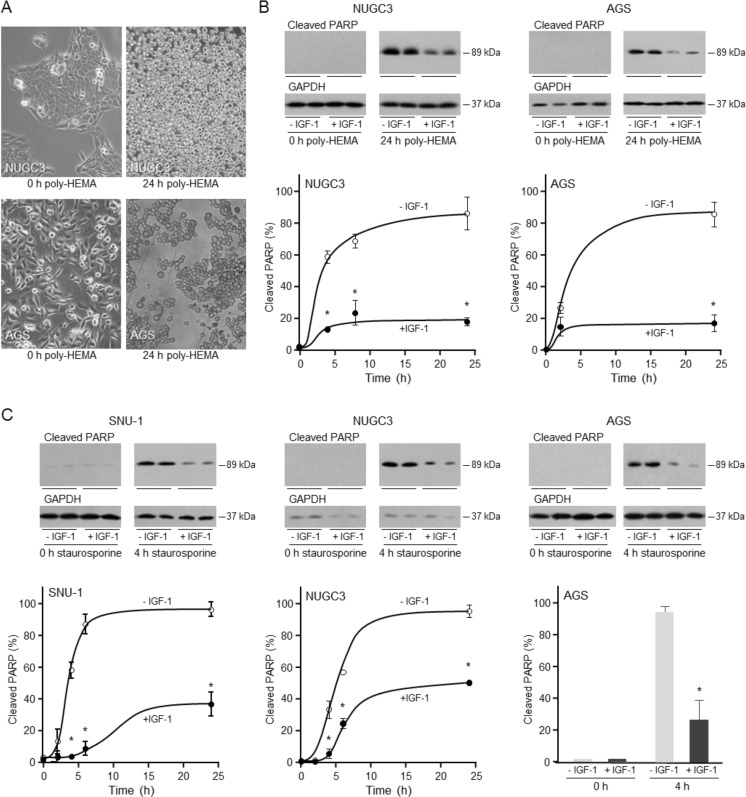
Protective effect of IGF-1 against anoikis and apoptosis in triple-negative gastric cancer cells For induction of anoikis, NUGC3 and AGS cells were added to uncoated or poly-HEMA-coated plates **A.** or to poly-HEMA-coated plates in the absence or presence of 50 ngml^−1^ IGF-1 **B.** Cells cultured in poly-HEMA-coated plates lost their characteristic polygonal appearance and grew as rounded detached cells **A.** Cells were lysed and the amount of cleaved PARP was analysed by Western transfer **B.** The amount of cleaved PARP was measured by densitometric scanning of the X-ray films, corrected for GAPDH expression with Labworks 4 software and expressed as the percentage of the maximum value measured for each cell line. The mean values ± SEM are shown. Asterisks indicate times at which cleaved PARP levels are statistically significantly lower in the presence of IGF-1 than in its absence (Two-way ANOVA; NUGC3, *p* < 0.0001; AGS, *p* < 0.0001). For apoptosis, SNU-1, NUGC3 and AGS cells were incubated with staurosporine in the absence and presence of 50 ngml^−1^ IGF-1 **C.** Cells were lysed and cleaved PARP measured and analyzed as described above (Two-way ANOVA; SNU-1, *p* < 0.0001; NUGC3, *p* < 0.0001; AGS, *p* = 0.0002).

To test the hypothesis that IGF-1 is important for the resistance of triple-negative gastric cancer cells to anoikis, cell attachment was prevented by culture in poly-HEMA-coated plates (Figure 3A) [31, 32]. The cells lost their characteristic polygonal morphology and appeared as rounded cells in suspension. Cleaved PARP was detected by 4 h and increased further by 24 h in unattached NUGC3 and AGS (Figure 3B). Incubation with IGF-1 reduced the amount of cleaved PARP four-fold and the amount remained low over 24 h. SNU-1 grow in suspension and are inherently resistant to anoikis and cell death was not induced in unattached MKN74 cells (data not shown).

Staurosporine is a protein kinase inhibitor that induces apoptosis via the intrinsic pathway [33]. In SNU-1, cleaved PARP was detected after two hours of staurosporine treatment and increased thereafter up to 24 hours (Figure 3C). IGF-1 protected SNU-1 cells from apoptosis. Similarly, IGF-1 prevented apoptosis in NUGC3 and AGS by up to 70%. Staurosporine did not initiate apoptosis in MKN74 which were remarkably resistant to cell death (data not shown).

**Figure 4:**
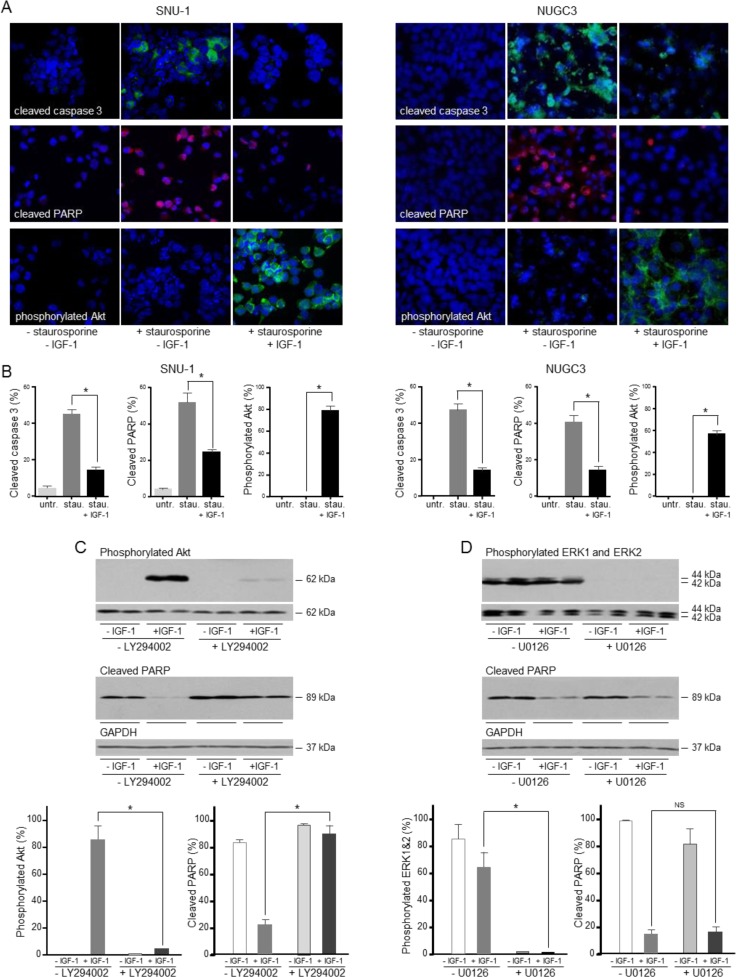
IGF protects gastric cancer cells from caspase-dependent cell death *via* the PI3-kinase/Akt pathway SNU-1 and NUGC3 cells were treated for 4 and 24 h, respectively with staurosporine (stau.) in the absence and presence of 50 ngml^−1^ IGF-1. Cells were fixed and incubated with antibodies against cleaved caspase 3, cleaved PARP and phosphorylated Akt **A.** and **B.** Nuclei were identified with the DAPI DNA dye. The proportion of cells with detectable cleaved caspase 3, cleaved PARP and phosphorylated Akt is shown as means ± SEM. Asterisks indicate differences that are statistically significant (One-way ANOVA; SNU-1, cleaved caspase 3, *p* < 0.0001; cleaved PARP, *p* = 0.0002; phosphorylated Akt, *p* < 0.0001; NUGC3, cleaved caspase 3, *p* < 0.0001; cleaved PARP, *p* < 0.0001; phosphorylated Akt, *p* < 0.0001). SNU-1 cells were treated with staurosporine in the absence and presence of 50 ngml^−1^ IGF-1 and 20 μM LY294002 or 6 μM U0126 inhibitor, lysed and cleaved PARP, phosphorylated Akt, ERK1 and ERK2 were measured and corrected for the expression of GAPDH or total corresponding protein **C.** Asterisks indicate phosphorylated protein levels that are significantly lower in the presence of an inhibitor than in its absence (Two-way ANOVA; phosphorylated Akt, *p* < 0.0001; phosphorylated ERK1 and ERK2, *p* = 0.0006) or cleaved PARP levels that are statistically significantly higher in the presence of an inhibitor (Two-way ANOVA; for LY294002 inhibitor, *p* = 0.0001. NS indicates values that are not significantly different.

To confirm that the cell death induced is caspase-dependent, cleaved caspase 3 and cleaved PARP were analyzed by immunofluorescence. There was a significant increase in the proportion of SNU-1 and NUGC3 cells with detectable cleaved caspase 3 in staurosporine-treated compared to untreated cells (Figure 4A and 4B). IGF-1 reduced the proportion of SNU-1 and NUGC3 cells with detectable cleaved caspase 3. The increase in cleaved PARP detected after staurosporine treatment was reduced significantly by IGF-1 in SNU-1 and NUGC3.

Phosphorylated Akt was not detected in untreated cells or in apoptotic cells but was detected in the majority of staurosporine-treated SNU-1 and NUGC3 cells that had been incubated with IGF-1. The inference that activation of the PI3-kinase/Akt pathway may be important for the protective effect of IGF-1 on cell survival was tested with the PI3-kinase inhibitor, LY294002. IGF-stimulated phosphorylation of Akt was reduced significantly in the presence of LY294002 and there was a concomitant abrogation of the protective effect of IGF-1 on cell survival (Figure 4C). In contrast, while the MEK1 and MEK2 inhibitor, U0126, prevented phosphorylation of ERK1 and ERK2, it had no effect on IGF-protection (Figure 4D). These results indicate that activation of the PI3-kinase/Akt pathway is important for IGF-1-protection against cell death.

**Figure 5:**
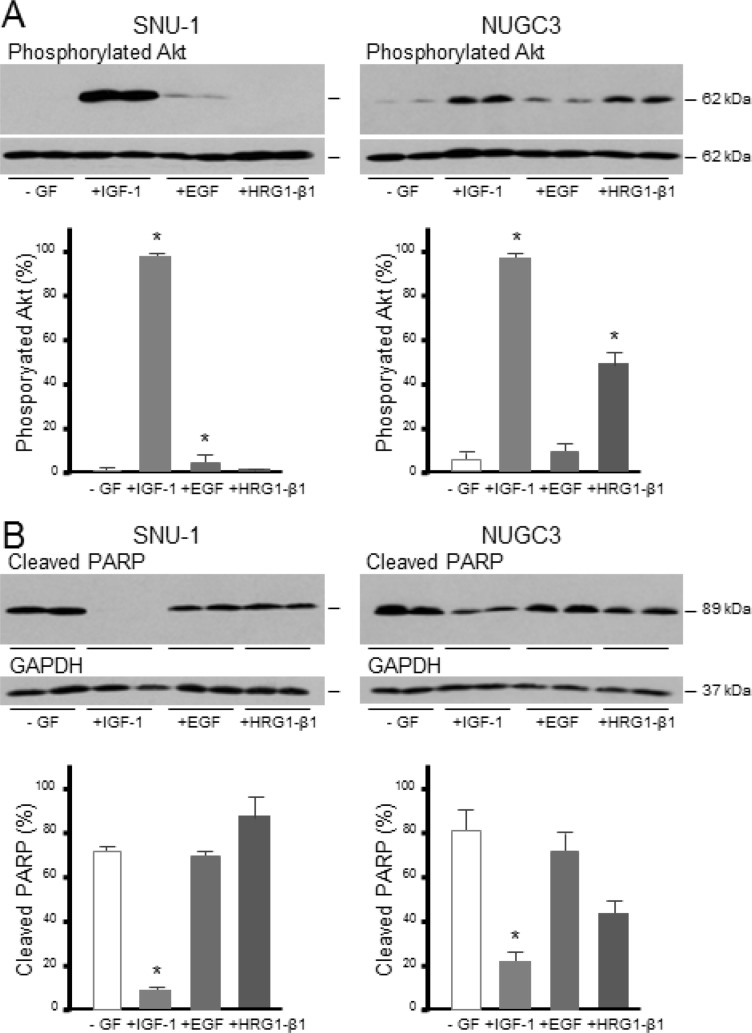
IGF, but not EGF or heregulin, protects gastric cancer cells from caspase-dependent cell death SNU-1 and NUGC3 cells were incubated with staurosporine in the absence (−GF) and presence of 50 ngml-1 IGF-1, EGF or HRG1-β1. Phosphorylated Akt, total Akt, cleaved PARP and GAPDH were measured and analyzed as described in the legends to figures 3 and 4 **A.** and **B**. Asterisks indicate differences that are statistically significant (One-way ANOVA; cleaved PARP, SNU-1 IGF-1, *p* < 0.0001; NUGC3, IGF-1, *p* < 0.01).

To investigate if triple-negative gastric cancer cells are protected from apoptosis by other growth factors, cells were incubated with IGF-1, epidermal growth factor (EGF) or the EGF domain of heregulin-β1 (HRG1-β1). In SNU-1, IGF-1 induced massively phosphorylation of Akt, EGF induced some phosphorylation but HRG1-β1 did not stimulate detectable phosphorylation of Akt (Figure 5A). IGF-1 was the only growth factor that protected SNU-1 cells from anoikis (Figure 5B). In NUGC3, both IGF-1 and HRG1-β1 stimulated Akt phosphorylation but only IGF-1 protected cells from apoptosis.

### The IGF signal transduction pathway in patient samples

**Figure 6:**
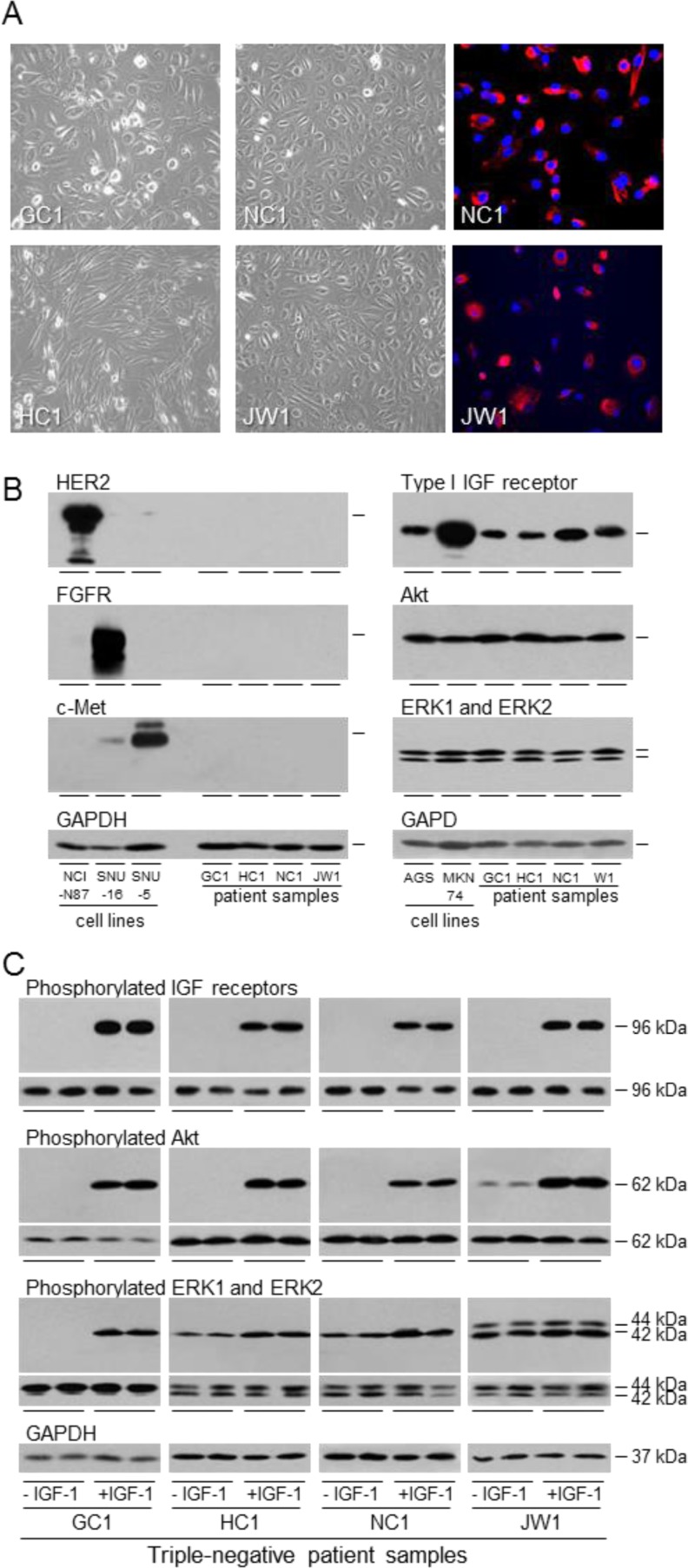
Expression and activation of the IGF signal transduction pathway in patient samples GC1, HC1, NC1 and JW1 cells were grown to 70% confluence and photographed or fixed and incubated with fluorescently-labelled antibody against epithelial cytokeratins **A.** Expression of HER2, FGFR2, c-Met, type I IGF receptor, Akt, ERK1 and ERK2 were analyzed by western transfer as described in the legend to Figure 1 **B.** GC1, HC1, NC1 and JW1 cells were withdrawn for two days and stimulated with 50 ngml^−1^ IGF-1 for 15 min. Phosphorylation of IGF receptors, Akt, ERK1 and ERK2 was analyzed by western transfer as described in the legend to Figure 2**A.**

Malignant gastric cancer cells were isolated from the ascitic fluid accumulated in the peritoneal cavity of patients with advanced gastric adenocarcinoma. The morphology of cells following isolation is shown in Figure 6A. Tumour cells grew with a characteristic epithelial-cell, pavement-like appearance as monolayers in culture and expressed epithelial cytokeratins. None of the patient cells expressed detectable HER2, FGFR2 or c-Met consistent with absence of *HER2-, FGFR2-* and *Met*-amplification (Figure 6B). In contrast, expression of the type I IGF receptor was detected. Incubation in the absence and presence of IGF-1 demonstrated IGF-stimulated receptor activation by auto-phosphorylation (Figure 6C). IGF-1 stimulated dramatically phosphorylation of Akt in all the patient samples. ERK1 and ERK2 phosphorylation was stimulated in all cells and increased markedly in GC1. These malignant cells isolated directly from gastric cancer patients have clearly a functional and responsive IGF signal transduction pathway.

**Figure 7:**
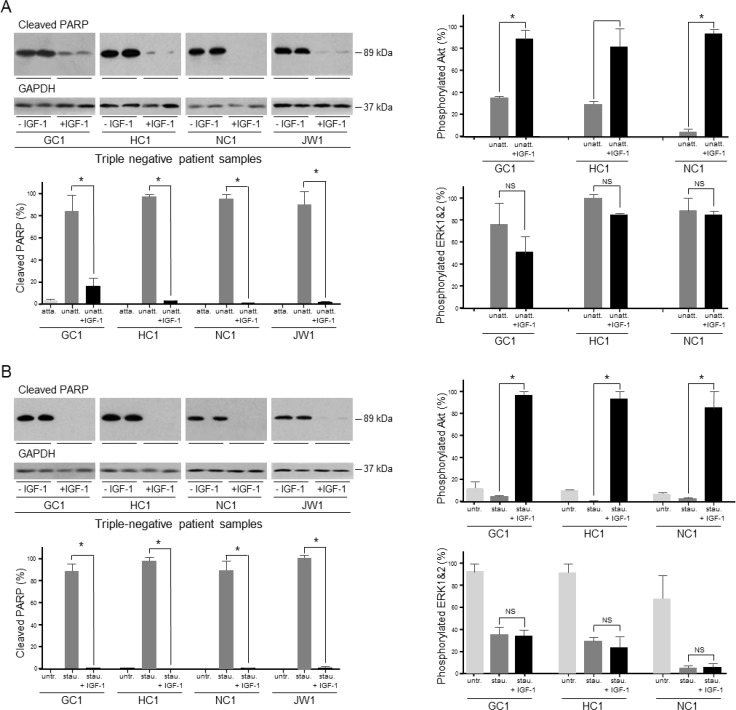
Role of IGF signal transduction in the survival of gastric cancer patient samples induced to undergo anoikis or apoptosis GC1, HC1, NC1 and JW1 cells were grown in poly-HEMA-coated wells, in the absence or presence of 50 ngml^−1^ IGF-1 for 24 h (GC1 and HC1) or 6 h (NC1 and JW1) **A.** Cells were incubated in the absence or presence of 0.5 μM staurosporine (GC1, HC1 and JW1) or 1 μM staurosporine (NC1), in the absence or presence of 50 ngml^−1^ IGF-1 for 5 h **B.** Cells were lysed and cleaved PARP, GAPDH, phosphorylated Akt, ERK1 and ERK2 were measured. Asterisks indicate levels that are statistically significantly lower or higher in the presence of IGF-1 than in its absence (One-way ANOVA; for anoikis GC1; cleaved PARP, *p* < 0.0001, pAkt, *p* < 0.001, HC1; cleaved PARP, *p* = 0.0003, NC1, cleaved PARP, *p* < 0.0011, pAkt, *p* = 0.0003; JW1, cleaved PARP, *p* < 0.0004; for apoptosis; GC1; cleaved PARP, *p* < 0.0001, pAkt, *p* < 0.001, HC1; cleaved PARP, *p* < 0.0001, pAkt, *p* < 0.0001, NC1, cleaved PARP, *p* < 0.0001, pAkt, *p* = 0.0033; JW1, cleaved PARP, *p* < 0.0001). NS indicates values that are not significantly different.

Anoikis was induced in patient samples by culture in poly-HEMA-coated wells to prevent attachment. The cells became rounded and PARP cleavage was detected. Incubation in the presence of IGF-1 conveyed anoikis resistance (Figure 7A). Concomitant with the IGF-protection of the unattached cells from anoikis, IGF-1 stimulated activation of the PI3-kinase/Akt but not the Ras/Raf/MAP-kinase pathway which is consistent with the survival effect being mediated through the PI3-kinase/Akt pathway.

The protective effect of IGF-1 against induction of apoptosis was tested in the patient samples. PARP cleavage was induced, and IGF-1 shown to reduce PARP cleavage to barely detectable levels in these metastatic tumor cells (Figure 7B). As with protection from anoikis, the protection afforded against apoptosis was greater for the patient cells than for the established gastric cancer cell lines. IGF-1 stimulated significant phosphorylation of Akt even in the presence of staurosporine. In contrast, IGF-1 did not stimulate activation of the Ras/Raf/MAP-kinase pathway in staurosporine-treated cells.

### Importance of the IGF signal transduction pathway in proliferation

**Figure 8:**
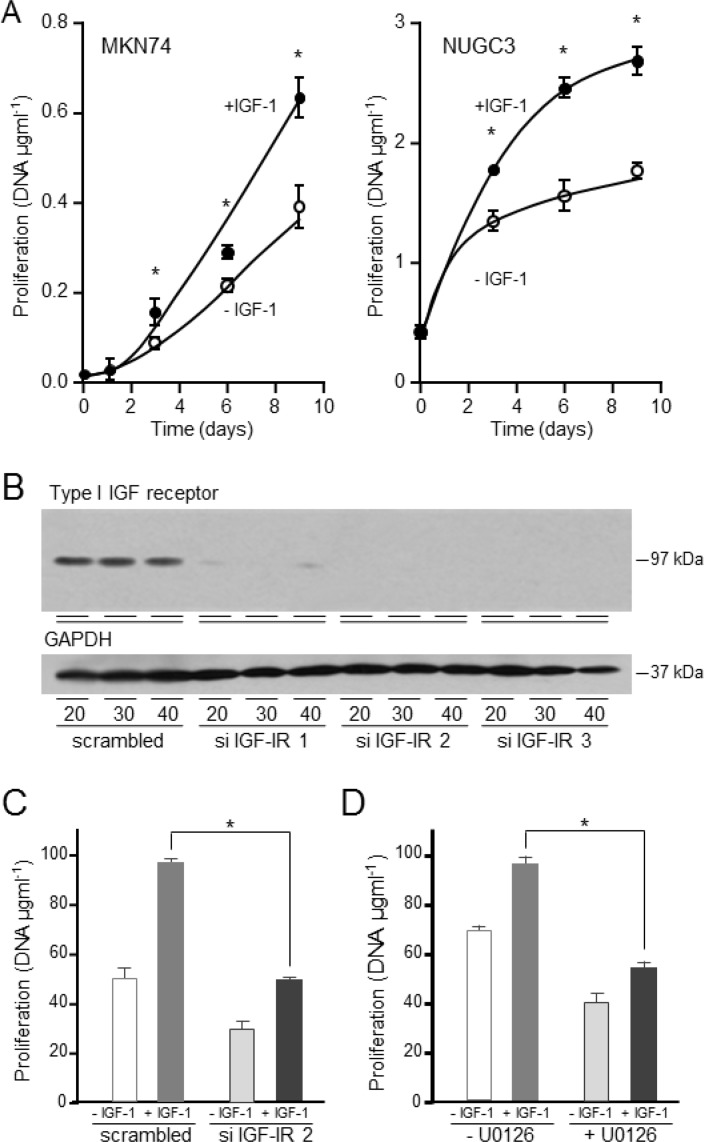
IGF-1 stimulates gastric cancer cell proliferation *via* the type I IGF receptor and Ras/Raf/MAP-kinase pathway MKN74 and NUGC3 cells were plated in 16-mm-diameter wells, incubated in the absence or presence of 50 ngml^−1^ IGF-1, lysed and their DNA content measured **A.** Asterisks indicate times at which there were significantly more cells in the presence of IGF-1 than in its absence (Two-way ANOVA; MKN74, *p* < 0.0001; NUGC3, *p* < 0.0001). NUGC3 cells were transfected with 20, 30 or 40 nM of a scrambled oligonucleotide or siRNA oligonucleotides against the type I IGF receptor, cultured for three days and type I IGF receptor and GAPDH expression analysed **B.** NUGC3 cells were transfected with either a scrambled oligonucleotide or si IGF-IR 2 and cultured in the absence or presence of 50 ngml^−1^ IGF-1 for 9 days, **C.** NUGC3 cells were incubated with 6 μM U0126 inhibitor, in the absence or presence of IGF-1 for 9 days **D.** Asterisks indicate statistically significant reduction in IGF-stimulated proliferation after receptor knockdown (Two-way ANOVA; *p* < 0.0001) or after incubation with U0126 (Two-way ANOVA; *p* < 0.0001).

IGF-1 increased significantly the growth of MKN74 by 50% and the growth rate of NUGC3 more than doubled in the presence of IGF-1 (Figure 8A). The role of the type I IGF receptor in the proliferative response to IGF-1 was investigated. Three siRNA sequences against the type I IGF receptor reduced its expression; two to undetectable levels (Figure 8B). The proliferative response to IGF-1 was reduced significantly in cells in which expression of the type I IGF receptor had been decreased (Figure 8C). Treatment of cells with the MEK1 and MEK2 inhibitor, U0126, which did not inhibit the IGF-cell survival effect, abrogated the proliferative effect of IGF-1 (Figure 8D). The data indicate that IGF-1 stimulated proliferation of gastric cancer cells is mediated through the type I IGF receptor and involves activation of the Ras/Raf/MAP-kinase pathway.

**Figure 9:**
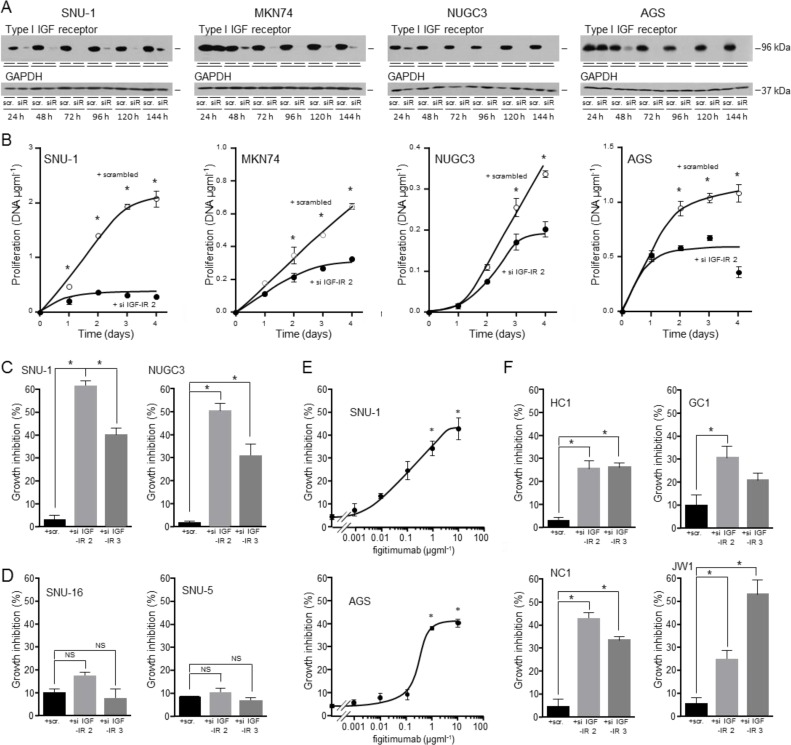
Importance of the type I IGF receptor in the growth of triple-negative gastric cancer cells and patient samples SNU-1, MKN74, NUGC3 and AGS cells were transfected with scrambled oligonucleotide (scr.) or si IGF-IR 2 (siR), cultured in DMEM and 10% FCS, lysed and type I IGF receptor and GAPDH **A.** or DNA content measured **B.** Asterisks indicate times at which there were significantly fewer cells after transfection with si IGF-IR 2 than with scrambled oligonucleotide (Two-way ANOVA; SNU-1, *p* < 0.0001; MKN74, *p* < 0.0001; NUGC3, *p* < 0.0001; AGS, *p* < 0.0001). SNU-1 and NUGC3 **C.**, and SNU-5 and SNU-16 cells **D.** were transfected with scrambled oligonucleotides, si IGF-IR 2 or si IGF-IR 3 and cultured for 4 days, lysed and their DNA content measured. Asterisks indicate significant growth inhibition after reduction in receptor expression (One-way ANOVA; *p* < 0.001). NS indicates values that are not significantly different. SNU-1 and AGS cells were cultured in the presence of the indicated concentrations of the IGF inhibitory antibody figitumumab for 4 days. Cells were lysed and their DNA content measured. Asterisks indicate significant growth inhibition in the presence of figitumumab (One-way ANOVA; *p* < 0.001). GC1, HC1, NC1 and JW1 cells were transfected with scrambled oligonucleotides, si IGF-IR 2 or si IGF-IR 3 and cultured for 7 days in DMEM and 20% FCS **E.** Cells were lysed and their DNA content measured. Asterisks indicate significant growth inhibition after reduction in receptor expression (One-way ANOVA; HC1, *p* < 0.001; GC1, *p*= 0.0322; NC1, *p* < 0.0001; JW1, *p* = 0.0006).

The overall importance of the type I IGF receptor in the growth of the gastric cancer cells was tested by its knockdown in cells cultured in the presence of serum (Figure 9A). Reduction of type I IGF receptor expression had a dramatic effect on growth (Figure 9B). The effect was most rapid and most marked in *KRAS*-mutant SNU-1 for which significant growth inhibition occurred within 24 h; inhibition was maintained for the duration of the experiment. Growth of *BRAF*-impaired MKN74, and *KRAS-* and *PI3KCA*-mutant AGS was reduced significantly two days after transfection and of NUGC3 cells after three days. None of the gastric cancer cells in which the type I IGF receptor had been knocked down grew significantly between days 3 and 4.

The importance of the type I IGF receptor was confirmed by knockdown with another siRNA that prevents receptor expression (si IGF-IR 3; Figure 8B). In triple-negative SNU-1 and NUGC3, knockdown of type I IGF receptor expression with both si IGF-IR 2 and si IGF-IR 3 reduced significantly cell growth. Proliferation of cells that overexpress HER2, FGFR2 or c-Met is expected to be driven through these receptors and to be less affected by expression of type I IGF receptor. Knockdown of the type I IGF receptor had no effect on *FGFR2*-amplified SNU-16 or *MET*-amplified SNU-5 cell growth (Figure 9D). The absence of a significant effect on the proliferation of SNU-16, or SNU-5 which express extremely low amounts of type I IGF receptor, corroborates the significance of the finding with the triple-negative cells. Orthogonal evidence for the importance of the type I IGF receptor was sought by pharmacological inhibition with the specific antibody, figitumumab. There was a clear concentration-dependent reduction in SNU-1 and AGS cell growth after culture in the presence of figitumumab (Figure 9E).

The contribution of the type I IGF receptor to the growth of gastric cancer cells isolated from patients was investigated. The tumor cells were transfected with scrambled oligonucleotides or type I IGF receptor si IGF-IR 2 or si IGF-IR 3 and incubated in medium that contained 20% serum. Growth of the patient cells was decreased between 30% and 60% after receptor knockdown (Figure 9F).

**Figure 10:**
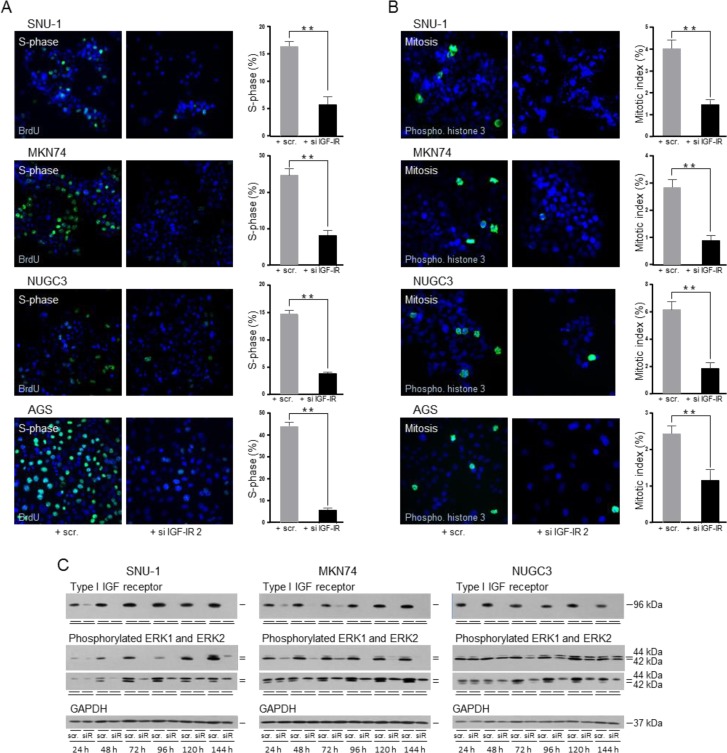
Abrogation of the type I IGF receptor prevents progression through the cell cycle and inhibits Ras/Raf/MAP-kinase pathway activity SNU-1, MKN74, NUGC3 and AGS cells were transfected, plated onto coverslips and cultured in serum-containing medium for three days. Cells were assayed for BrdU incorportation **A.** or histone H3 phosphorylation **B.** SNU-1, were processed in suspension. Representative photomicrographs and the proportion of cells in the S-phase or the mitotic-phase of the cell cycle are shown; asterisks indicate that the proportion of cells in either phase of the cell cycle is significantly lower after transfection with si IGF-IR 2 than with the scrambled oligonucleotide (Unpaired *t*-test; S-phase: SNU-1, *p* = 0.0078; MKN74, *p* = 0.0001; NUGC3, *p* = 0.0001; AGS, *p* = 0.0004 and mitotic-phase; SNU-1, *p* = 0.0009; MKN74, *p* = 0.0046; NUGC3, *p* = 0.0023; AGS, *p* = 0.017). SNU-1, MKN74 and NUGC3 cells were transfected with scrambled oligonucleotide (scr.) or si IGF-IR (siR), cultured in serum-containing medium, lysed and type I IGF receptor, phosphorylated ERK1 and ERK2, total ERK1 and ERK2 and GAPDH were measured (C).

To confirm that the reduction of cell number resulted from decreased cell proliferation, we analyzed cell cycle progression three days after transfection when growth was ceased completely. BrdU incorporation into newly synthesized DNA identifies cells in S-phase. There was a significant reduction, in the proportion of cells in which BrdU incorporation was detected after knockdown of the type I IGF receptor in all four triple-negative gastric cancer cell lines (Figure 10A). Phosphorylation of histone H3 on Ser10 occurs during chromosome condensation [34] and its detection discriminates cells in mitosis. The proportion of cells undergoing mitosis was reduced significantly in cells transfected with type I IGF receptor siRNA compared to in cells transfected with scrambled oligonucleotide (Figure 10B).

These data indicate that gastric cancer cell growth is responsive to IGF-1 and that the type I IGF receptor is critical for progression through the cell cycle. The question of how removal of the receptor could be so effective in *KRAS*-activated SNU-1 remained and we investigated the effect of receptor knockdown on activation of the Ras/Raf/MAP-kinase pathway (Figure 10C). Phosphorylation of ERK1 and ERK2 was reduced within 24 h after receptor knockdown in RAS-activated SNU-1 and in *BRAF*-impaired MKN74 and remained low up to 144 h after transfection. Phosphorylation of ERK1 and ERK2 was reduced also after transfection of wild-type NUGC3, albeit less strongly. Subsequent to the reduction in type I IGF receptor expression and ERK1 and ERK2 phosphorylation, reduction in total ERK1 and ERK2 was detected after 48 h and was maintained until day six.

## DISCUSSION

Amongst the gastric cancer cell lines analyzed, NCI-N87 expressed high levels of HER2, KATO III and SNU-16 expressed high levels of FGFR2 and SNU-5 overexpressed c-Met consistent with reported amplification of the genes that encode these tyrosine kinase receptors [35]. Kato III and SNU-16 have low-level copy number increase in *MET* due, in Kato III, to aneuploidy of chromosome 7 [36]. SNU-1, MKN-74, NUGC3 and AGS represent the majority of gastric cancers that do not overexpress the three receptors against which drugs are licensed or in clinical trial. Importantly, we show that they express the type I IGF receptor. The type I IGF receptor was expressed also in gastric cancer cells isolated directly from patients that do not express HER2, FGFR2 or c-Met.

IGF-1 stimulated phosphorylation of the type I IGF receptor and Akt in all the gastric cancer cell lines analyzed which demonstrates that the IGF signal transduction pathway is active in cells that overexpress *HER2, FGFR2* or *MET*. A recent study of NCI-N87 suggested that activity of the IGF signal transduction pathway confers lapatinib resistance [18]. Interestingly, IGF-1 stimulated Akt phosphorylation in *FGFR2*-amplified Kato III and SNU-16 and MAPK phosphorylation in SNU-16. This is the first demonstration that the IGF signal transduction pathway is active in *FGFR2*-amplified gastric cancer cells. Loss of *IGF1R* expression did not inhibit the growth in full medium of *FGFR2*-amplified SNU-16 but it is possible that the active IGF pathway might acquire ascendancy after abrogation of FGFR pathway activity. Our findings that IGF-1 stimulates phosphorylation of the type I IGF receptor and Akt in *MET*-amplified SNU-5 are consistent with reports that IGF-1 increases trypan blue uptake and protects against cell death induced with 5% ethanol [16] in *MET*-amplified MKN45 [36], and that an shRNA against the type I IGF receptor increased MKN45 cell death and decreased colony formation in soft agar [17]. The IGF signal transduction pathway may be more important than appreciated in cells with amplified *HER2, FGFR2* or *MET* and consideration should be given to dual targeting with an IGF inhibitor to limit onset of resistance.

The main aim of our study was to test the hypothesis that *IGF1R*, and the downstream IGF signal transduction pathway and phenotypic response [37], is important and hence a viable therapeutic target in gastric cancer cells that are not addicted to *HER2, FGFR2* or *MET* and are ineligible for *HER2-, FGFR-* or *MET*-targeted therapies. We demonstrated a cell survival effect of IGF-1 against caspase-dependent apoptosis induced by anchorage deprivation or by protein kinase inhibition. The protective effect of IGF-1 was associated with activation of the PI3-kinase/Akt pathway but not the Ras/Raf/MAP-kinase pathway, and was prevented by inhibitors of the former but not the latter pathway. That IGF-1 protects against apoptosis induced by the kinase inhibitor staurosporine emphasizes the potency of IGF-1 as a pro-survival factor and suggests that it may protect gastric cancer cells from therapeutic kinase inhibitors. The resistance to cell death induction of MKN74 contrasts with a previous report that cell death was induced by 5% ethanol [16].

The prosurvival effect of IGF-1 was more pronounced for gastric cancer cells isolated directly from patients than for cell lines which shows that IGF-dependence is an inherent characteristic of gastric cancer cells and is not acquired during culture. The concomitant activation of the PI3-kinase/Akt but not the Ras/Raf/MAP-kinase pathway indicates that the cell survival signal is transmitted via the former pathway. The role of IGF-1 in protection against anoikis is of particular interest because circulating IGFs could promote survival of detached gastric cancer cells present in serum or ascitic fluid and hence increase their metastatic potential.

Proliferation of gastric cancer cells was stimulated by IGF-1 and the stimulation was decreased by inhibition of the Ras/Raf/MAP-kinase pathway. Consistent with the abrogation of IGF-stimulated growth by MEK1 and MEK2 inhibition, ERK1 and ERK2 phosphorylation was reduced concomitantly with the decreased cell growth observed after reduction in type I IGF receptor expression. The inhibition of proliferation and ERK1 and ERK2 phosphorylation were more pronounced in the *KRAS*-mutant SNU-1 and *BRAF*-impaired MKN-74 than in wild-type NIGC3. Subsequent to the reduction in type I IGF receptor expression and ERK1 and ERK2 phosphorylation, less ERK1 and ERK2 were detected. ERK1 and ERK2 are targeted for proteasomal degradation following ubiqutination by the E3 ubiquitin ligase activity of the PHD domain of MEKK1 [38]. Removal of IGF signal transduction may potentiate this effect. Alternatively, IGF inhibition may exacerbate the caspase cleavage and inactivation of ERK2 which reduces the half-life of ERK2 and is associated with p53-mediated growth arrest in RAS-mutant cells [39]. That MAP-kinase activation is important for IGF-1 stimulated proliferation indicates distinct roles for Akt and MAP-kinase activation in response to IGF-1. Activation of the PI3-kinase/Akt, but not the Ras/Raf/MAP-kinase pathway, is required for the survival effect of IGF-1 whereas MAP-kinase phosphorylation is required for its effect on proliferation.

Our results indicate that the type I IGF receptor mediates the IGF-1 proliferative effect. The importance of the type I IGF receptor in gastric cancer cell proliferation was shown by the significant reductions in cell growth in serum-containing medium and in the proportion of cells in S- and mitotic-phases of the cell cycle after knockdown of receptor expression. Demonstration that figitumumab reduced cell proliferation provides corroboration with an orthogonal approach of the importance of the type I IGF receptor in triple-negative gastric cancer cell growth. Growth inhibition was observed also after knockdown of the type I IGF receptor in gastric cancer cells isolated from patients. Previously, reduced colony formation has been shown after inhibition of the type I IGF receptor with shRNA and the αIR3 antibody in *MET*-amplified MKN45 and in tumour explants, respectively [17, 40]. Our data provide the first demonstration of the importance of the type I IGF receptor in the proliferation of triple-negative gastric cancer cells. The reduction in growth after type I IGF receptor knockdown and pharmacological inhibition indicates that loss of activity of this receptor cannot be compensated via activation of other receptors by growth factors present in the serum.

Proliferation of *KRAS*-G12D-mutant SNU-1 gastric cancer cells [41, 42] was abrogated by knockdown of the type I IGF receptor and reduced by its pharmacological inhibition. These observations concur with the demonstration that IGF receptor inhibitors have selectivity for *KRAS*-mutant lung cancer cells [43]. The authors showed that *KRAS*-mutant NSCLC cells depend upon the IGF signal transduction pathway to provide an initial stimulus to the Ras/Raf/MAP-kinase pathway and concluded that therapeutic inhibition of the IGF signal transduction pathway could be particularly effective in *KRAS*-mutant lung cancer. A similar requirement for activation of the IGF pathway has been demonstrated for *K*RAS-mutant colorectal cancer cells [44]. There seems to be a delicate balance in established cancers with oncogenic RAS mutations between adequate primary activation of the Ras/Raf/MAP-kinase pathway and evasion of cell senescence consequent to strong negative feedback through mTORC1 and S6K [45, 46]. The balance is achieved best if the primary stimulus is through a receptor such as the type I IGF receptor that activates preferentially the PI3-kinase/Akt pathway [43, 44]. The pronounced reduction in phosphorylation of ERK1 and ERK2 after knockdown of the type I IGF receptor in *KRAS*-mutant SNU-1 gastric cancer cells is consistent with primary activation of their Ras/Raf/MAP-kinase pathway being dependent upon stimulus through the IGF signal transduction system. Patients with *KRAS*-mutant colorectal cancer do not benefit from EGFR-targeted therapy [47] and outgrowth of KRAS-mutant cells accompanies onset of resistance [48]. Similarly, *KRAS* and *HRAS* mutations confer resistance to MET-targeted agents [49]. These findings imply that, unlike the effective co-existence of *KRAS*-mutations and signal transduction through the type I IGF receptor, signal transduction *via* EGFR or c-Met cannot complement *KRAS*-mutations in established malignant cells.

MKN74 have a *BRAF*-impaired mutation which substitutes Gly466 with a valine residue and are insensitive to vemurafenib [30]. Gly466 lies within the glycine-rich P-loop of B-Raf and is involved in stabilization of the inactive conformation of the kinase domain via hydrophobic interactions with Leu597 and Val600 [50]. B-Raf G466V lacks auto-inhibition but is recruited to the plasma membrane to interact with Ras, form heterodimers with c-Raf and, although its kinase domain is inactive, act as a scaffold protein to enhance c-Raf activity and hence hyper-activation of the Ras/Raf/MAP-kinase pathway [51, 52]. Our demonstration that type I IGF receptor knockdown prevents proliferation of *BRAF*-impaired MKN74 gastric cancer cells suggests that *BRAF*-impaired cells may have a similar dependency upon activation of IGF signal transduction to that reported for *RAS*-mutant cells [43, 44]. To our knowledge, this is the first report that Raf-impaired cancer cells may have such reliance upon IGF signal transduction. Our findings concur with the demonstration that in oncogenic *RAS*-mutant and kinase-dead *BRAF*-mutant cells, tumor progression is driven through a similar mechanism of c-Raf recruitment and hyper-activation [53] and suggest that *BRAF*-impaired cancer cells may be as susceptible to therapeutic inhibition of IGF signal transduction as has been proposed for *RAS*-mutant cells [43, 44].

In conclusion, *IGF1R* expression and the IGF signal transduction pathway are important in the survival and proliferation of triple-negative gastric cancer cells. Reduction in IGF signal transduction may be required for targeted therapeutic strategies to be effective in patients with triple-negative gastric cancers including those with Ras-addicted or Raf-impaired cancers.

## MATERIALS AND METHODS

### Cell culture

Gastric cancer cell lines, NCI-N87, KATO III, SNU-16, SNU-5, SNU-1, MKN74, NUGC3 and AGS were purchased from American Type Culture Collection (Manassas, VA) or DMCSZ and cultured in Dulbecco's modified Eagle's medium and 10% fetal calf serum (FCS). NCI-N87, MKN74, NUGC3 and AGS are adherent and SNU-1, SNU-5 and SNU-16 grow in suspension. KATO III grow as a mix of attached and non-attached cells. Cells were maintained in exponential growth at 37°C in a humidified atmosphere, with 5% CO_2_.

Ethical permission was obtained from the Joint Newcastle Health Hospitals and University of Newcastle upon Tyne Ethical Committee. All patients gave informed consent. Ascites was collected from metastatic gastric cancer patients who were symptomatic for ascitic fluid accumulation and were having their ascitic fluid drained to alleviate their symptoms. A sample of the ascitic fluid was analyzed, and examined by a consultant cytologist to confirm presence of malignant cells. The ascitic fluid was diluted 1:2 in DMEM with 20% FCS and cultured at 37°C in a humidified atmosphere, with 5% CO_2_. Subsequently, cells were cultured in DMEM and 20% FCS as above and assessed for expression of epithelial growth factor receptor and epithelial cytokeratins by western transfer analysis and immunofluorescence, respectively.

### Western transfer analysis

Cells were lysed in radioimmunoprecipitate (RIPA) buffer, 50 mM Tris-HCl pH 7.5, 150 mM NaCl, 1 mM EDTA, 1% NP-40 (v/v), 0.25% sodium deoxycholate (w/v), 1 μgml^−1^ pepstatin, 1 μgml^−1^ aprotinin, 1 μgml^−1^ leupeptin, 2 mM sodium orthovanadate, 2 mM sodium fluoride and 2 mM phenyl methyl sulphonyl fluoride [27]. Protein concentrations were measured by bicinchonic acid assay (Thermo Scientific, Loughborough, UK) [54]. Proteins were separated by polyacrylamide gel electrophoresis, transferred to 0.45-μm nitrocellulose and incubated with antibodies against type I IGF receptor (#3027), phosphorylated IGF receptors (#3024), HER2 (#2165), c-Met (#4560), Akt (#9272), phosphorylated Akt (#4060), ERK1 and ERK2 (#9102), phosphorylated ERK1 and ERK2 (#4370), cleaved PARP (#9541) (Cell Signaling Technologies, Hitchin, United Kingdom), FGFR2 (PA5-29246) (Thermo Fisher Scientific) and GAPDH (sc-25778) (Santa Cruz biotechnology, Heidelberg, Germany). Antibodies that detect type I IGF receptor auto-phosphorylated on tyrosines 1135 and 1136 detect insulin receptor auto-phosphorylated on tyrosines 1150 and 1151. The activated ERK-specific antibody detects ERK1 phosphorylated on Thr 202 or Tyr 204 and ERK2 phosphorylated on Thr 185 or Tyr 187. The activated Akt-specific antibody detects Akt1, Akt2 and Akt3 phosphorylated on Ser 473, Ser 474 and Ser 472, respectively. Membranes were incubated with horseradish peroxidase-conjugated secondary antibody, SuperSignal West Dura Substrate (Thermo Scientific) and exposed to X-ray film.

### Immunofluorescence

Cells were fixed in methanol, 70% ethanol or 4% paraformaldehyde and incubated with Alexa Fluor-conjugated antibodies against cleaved caspase 3 (#9603) cleaved PARP (#6894), pan cytokeratins (#3478) or unconjugated-antibodies against phosphorylated Akt (#4060), BrdU (#5292), Ser10-phosphorylated histone H3 (#9701) (Cell Signaling Technologies), followed by incubation with Alexa fluorochrome-conjugated antibodies (#A-11034, #A-11001) (Invitrogen, Paisley, United Kingdom). Cells were incubated with 0.03 mgml^−1^ BrdU for 2 h prior to incubation with BrdU antibody. Cells were washed, mounted in Vectashield Mounting Medium with DAPI (Vector laboratories) and visualized with a fluorescent microscope. Five fields of view were quantified for each value.

### siRNA knockdown

Synthetic double-stranded short interfering RNA (siRNA) sequences that target the type 1 IGF receptor or scrambled sequences (Sigma-Aldrich) were mixed with lipofectamine (Invitrogen) in DMEM and incubated for 30 min at room temperature. Cells were trypsinized, centrifuged and resuspended in DMEM with 10% FCS and added to the siRNA transfection mixture. Oligonucleotide final concentrations were between 20 and 50 nM. After incubation, cells were lysed for western transfer analysis, processed for immunofluorescence, or their cell proliferation or survival analyzed

### IGF-I-stimulated protein phosphorylation

Cells were added to 35-mm-diameter wells, allowed to attach for 24 hours and withdrawn from the stimulating effects of growth factors in serum by culture for two days or one day (SNU-1) in phenol red-free DMEM supplemented with 10% calf serum that had been incubated with dextran-coated charcoal-treated (DCCS) at 55°C [25, 28]. Cells were washed twice with phosphate buffered serum (PBS), and cultured in the withdrawal medium for 24 h after which the cells were rewashed twice in PBS and cultured in withdrawal medium for a further 24 h. Cells were washed twice in PBS, incubated in serum-free medium for 2 h, and then in the absence or presence of 50 ngml^−1^ IGF-1 in serum free-medium for 15 min. Cells were lysed in RIPA buffer and phosphorylated or total individual proteins were detected by western transfer.

### Cell survival

For the anoikis assay, cells were washed with PBS and cultured for two days in withdrawal medium as described above, trypsinized and 200,000 cells were added to 35-mm-diameter wells, pre-coated with non-ionic acid poly(2-hydroxyethyl methacrylate) (poly-HEMA; SIGMA) which inhibits matrix deposition and prevents cell attachment [32]. Cells were incubated for 0 to 24 h in the poly-HEMA-coated wells in the absence or presence of 50 ngml^−1^ IGF-1 and lysed in RIPA buffer.

For apoptosis, 150,000 cells were added to 35-mm-diameter wells or 22-mm-diameter coverslips, allowed to attach for 24 h and withdrawn from growth factors by culture in withdrawal medium for 2 days, as described above. Cells in suspension were grown until ~70% confluence and centrifuged at 300 x g for 3 minutes and resuspended in PBS. Cells were centrifuged again at 300 x g for 3 minutes and resuspended in withdrawal medium. After two days withdrawal, cells were incubated in withdrawal medium and 0.5-1 μM staurosporine (Sigma-Aldrich, Dorset, United Kingdom) for various times, lysed in RIPA buffer or fixed in methanol or paraformaldehyde. The protective effects of growth factors were tested by preincubation for 15 minutes in 50 ngml^−1^ IGF-1, EGF or HRG1-β1. Cells were incubated without and with 20 μM PI3-kinase inhibitor, LY294002, (Cell Signaling) or 6 μM MEK1 and MEK2 inhibitor, U0126, (Calbiochem) for 30 min prior to addition of IGF-1. Cell death was assessed by the extent of PARP or caspase 3 cleavage analyzed by western transfer or immunofluorescence.

### Cell proliferation

Aliquots of 5,000 cells were added to 16-mm-diameter wells and allowed to attach for 24 hours. MKN74 cells were cultured with 2% DCCS and NUGC3 cells in serum-free medium in the absence or presence of 50 ngml^−1^ IGF-1. Cells were incubated without and with 6 μM U0126, for 30 min prior to addition of IGF-1.

SNU-1, MKN74, NUGC3, AGS, SNU-16 and SNU-5 that had been transfected with siRNA oligonucleotides were cultured in DMEM and 10% FCS. GC1, HC1, NC1 and JW1 cells were cultured in DMEM and 20% FCS. SNU-1 and AGS cells were incubated without and with different concentrations of the type I IGF receptor antibody figitumumab. Cells were lysed and DNA content measured with Pico-Green dsDNA Quantitation Reagent (Invitrogen) [27].

### Statistics

For the western transfer images or immunofluorescence experiments, a representative example is shown. The optical density of protein bands was quantified by densitometric analysis with LabWorks 4.0 software, adjusted for the optical density of the X-ray film and corrected for GAPDH or total individual protein expression (UVP, Inc, Cambridge, United Kingdom). Data were normalized and expressed as a percentage of the maximum cleaved PARP or activated signal transduction protein detected. For each immunofluorescence replicate, the number of cells in which the molecule under analysis was detected was counted in five individual fields. Data were normalized for the maximum amount of the molecule detected in each experiment. Experiments were replicated at least thrice. Results are expressed as means ± S.E.M. Differences between groups were tested by one or two way analysis of variance, or unpaired t-test. P values < 0.05 were considered statistically significant.
